# Investigating the Chaperone Properties of a Novel Heat Shock Protein, Hsp70.c, from *Trypanosoma brucei*

**DOI:** 10.1155/2014/172582

**Published:** 2014-02-24

**Authors:** Adélle Burger, Michael H. Ludewig, Aileen Boshoff

**Affiliations:** Biomedical and Biotechnology Research Unit (BioBRU), Department of Biochemistry, Microbiology and Biotechnology, Rhodes University, P.O. Box 94, Grahamstown 6140, South Africa

## Abstract

The neglected tropical disease, African Trypanosomiasis, is fatal and has a crippling impact on economic development. Heat shock protein 70 (Hsp70) is an important molecular chaperone that is expressed in response to stress and Hsp40 acts as its co-chaperone. These proteins play a wide range of roles in the cell and they are required to assist the parasite as it moves from a cold blooded insect vector to a warm blooded mammalian host. A novel cytosolic Hsp70, from *Trypanosoma brucei*, TbHsp70.c, contains an acidic substrate binding domain and lacks the C-terminal EEVD motif. The ability of a cytosolic Hsp40 from *Trypanosoma brucei* J protein 2, Tbj2, to function as a co-chaperone of TbHsp70.c was investigated. The main objective was to functionally characterize TbHsp70.c to further expand our knowledge of parasite biology. TbHsp70.c and Tbj2 were heterologously expressed and purified and both proteins displayed the ability to suppress aggregation of thermolabile MDH and chemically denatured rhodanese. ATPase assays revealed a 2.8-fold stimulation of the ATPase activity of TbHsp70.c by Tbj2. TbHsp70.c and Tbj2 both demonstrated chaperone activity and Tbj2 functions as a co-chaperone of TbHsp70.c. *In vivo* heat stress experiments indicated upregulation of the expression levels of TbHsp70.c.

## 1. Introduction

Molecular chaperones are essential for the maintenance of cellular homeostasis by facilitating various functions including degradation of proteins [1], translocation [2], folding of co-translational products [3], and protein complex assembly [4]. Many molecular chaperones are also known as heat shock proteins (Hsp) and their expression is upregulated in response to stress. The large and varied heat shock protein family has usually been classified into major classes defined by molecular weight.

The 70 kDa family of proteins is the most abundant and thoroughly studied family of heat shock proteins [5]. Hsp70 functions both as a holdase (binding and holding onto unfolded polypeptides by withdrawing aggregation-prone species) as well as a refoldase (assisting non-native proteins to fold to the native state) [6]. The highly conserved Hsp70 structure is typically composed of an N-terminal ATPase domain (44 kDa), a C-terminal domain containing a *β*-sandwich subdomain, which is the substrate binding domain (15–18 kDa,) followed by an *α*-helical subdomain (10 kDa) [7, 8].

Hsp70 protein activity occurs through the interaction of hydrophobic peptide segments of other proteins with its substrate binding domain (SBD) in an ATP-dependent manner. The Hsp70 ATPase cycle switches between the low affinity ATP-bound state and rapid substrate-exchange rates and the high affinity ADP-bound state with low substrate-exchange rates [9]. Hsp40 is a co-chaperone and is required to stimulate the ATPase activity of Hsp70 and results in Hsp70-ADP with a high affinity for substrate. In order for the nucleotide binding cleft to be opened, the release of ADP is facilitated by a nucleotide-exchange factor, such as Bag-1 protein. The ATPase cycle is completed when ATP binds the ATPase domain of Hsp70 resulting in a conformational change in the substrate binding domain and bound substrates are released [10]. The EEVD motif present at the end of the C-terminus of Hsp70 members is involved in binding to the tetratricopeptide repeat (TPR) domains of Hsp70/Hsp90 organizing protein (Hop) and carboxy terminus of Hsp70 interacting protein (CHIP) [11]. The EEVD motif has been suggested to recruit Hsp40 as it has been shown to bind and regulate the function of Hsp40 [12, 13].

All Hsp40s contain a J-domain that is required for facilitating the cellular activity of Hsp70 proteins through its interaction with their partner Hsp70s. The J-domain has a conserved Histidine-Proline-Aspartic acid (HPD) tripeptide; variations within the HPD motif are known to abolish the stimulation of Hsp70 ATPase activity by Hsp40 [14, 15]. Hsp40s have been divided into three classes based on which functional domains they contain. Type I Hsp40s are highly conserved and contain a glycine-phenylalanine (G/F) and cysteine rich region that contains four motifs of CXXCXGXG, a glycine/methionine rich region, a carboxy-terminal peptide binding domain and a dimerization domain [16, 17]. Type I Hsp40s also have a peptide-binding region at the C-terminus which aids in the binding to non-native polypeptides, in turn allowing Hsp40 proteins to transport substrates to Hsp70 proteins for folding [18, 19]. Type II Hsp40s contain the J-domain and the G/F rich region, along with the peptide-binding region at the C-terminus [20–22]. Both Types I and II serve to bind substrates and target them to Hsp70 [22]. The highly specialized Type III Hsp40s possess only the conserved J-domain which can occur anywhere on its sequence [23] and primarily functions to recruit Hsp70 to a particular location [22].

The African trypanosome, *Trypanosoma brucei, *is a blood-borne unicellular parasitic protozoan and the etiological agent of both human and animal African Trypanosomiasis [24]. African Trypanosomiasis is described as one of the neglected tropical diseases crippling economic development and causing death in Africa's poorest and most marginalized communities. Due to a lack of new treatments against human African Trypanosomiasis and emergence of resistance to older drugs, human treatments currently available are connected with high levels of toxicity and resistance [25]. Heat shock proteins have been emerging as prospective drug targets (Hsp70s as drug targets reviewed in [26]). Drugs causing cellular stress resulting in the induction of heat shock proteins have been discovered to ultimately improve cytoprotection [27, 28]. Anti-heat shock protein drugs used in combination with current drugs could therefore synergistically improve the effectiveness of available drugs.

Parasitic heat shock proteins have been revealed as drug targets, including malarial drug target *P. falciparum* Hsp90 and trypanosomal *T. evansi* Hsp90 [29, 30]. Amongst the known Hsp90 proteins, PfHsp90 was shown to have the highest ATPase activity, and its inhibition by geldanamycin (GA) was stronger than seen for human Hsp90 [30]. Semi-synthetic inhibitor 17-(allylamino)-17-demethoxygeldanamycin (17-AAG) has been shown to curb growth of the *P. falciparum* parasite and to inhibit the growth of the *T. evansi* parasite by specifically binding and inhibiting Hsp90 [30]. 17-AAG could likely be effective against infection caused by *T. brucei* due to the sequence similarity between TbHsp90 and TeHsp90 [30]. Sera from patients infected with trypanosomes were screened to identify diagnostic antigens, and although TbHsp70 was identified as a candidate, by itself, it demonstrated inadequate specificity and sensitivity in diagnosis of trypanosomiasis [23]. It may however be useful as a diagnostic antigen if used in conjunction with other immunogenic proteins [23].

Of the Leishmanial and trypanosomal Hsp70s and Hsp40s, a number of important and well-known studies have been performed on *T. cruzi. *Cytoplasmic TcHsp70B demonstrated high ATPase activity in comparison to human Hsp70 [31] and was shown to be heat inducible [32]. In addition to stimulating the immune response [33], TcHsp70B is one of the immunodominant antigens in *T. cruzi* infected individuals [31, 33]. A Type I Hsp40, Tcj2, was able to stimulate the already high basal ATPase activity of TcHsp70B by 1.5 fold, whilst the Type III Hsp40 Tcj1 showed no significant stimulation [34]. Furthermore, Tcj2 was shown to complement the well-characterized *Saccharomyces cerevisiae *Type I Hsp40, Ydj1, and was subsequently suggested to be involved in cytoprotection [34]. TcHsp70B, displaying typical chaperone properties, is homologous to *T. brucei* Hsp70, and Tcj1 and Tcj2 are homologous to Tbj1 and Tbj2, respectively [35].

Genome annotation revealed* T. brucei* to possess a complement of 65 Hsp40 proteins and 12 Hsp70 proteins [35]. *In silico *analysis of TbHsp70.c indicated the presence of atypical acidic residues in the substrate binding domain as well as in the substrate binding cavity of TbHsp70.c [36] and these may play a role in substrate discrimination. However, the residues required for association with Hsp40 proteins within both the ATPase and substrate binding cavity, as well as the Hsp70 phosphorylation site, are well conserved. In addition the C-terminal EEVD motif is absent from TbHsp70.c and it contains the sequence RIEAINANTE at the end of the protein [36]. TbHsp70.c was predicted to be cytoplasmic [36]. To date, TbHsp70.c has not been biochemically characterized and co-chaperones have not been identified. Of the 65 Hsp40s, only one of the Type III *T. brucei* Hsp40 proteins, Tbj1, has been expressed, purified, and biochemically characterized to date [37]. No *in vitro *characterization of the Type I *T. brucei* Hsp40 protein, Tbj2, has been completed; *in vivo *analysis revealed that Tbj2 is essential to the survival of the cell [38, 39]. Tbj2 was demonstrated to be upregulated upon inducing heat shock and to localize in the cytoplasm [39]. According to TriTrypDB [40], TbHsp70.c is localized in the cytoplasm and Tbj2 was selected as a probable partner of TbHsp70.c. This research aims to functionally characterize TbHsp70.c and Tbj2 as its potential co-chaperone. We successfully expressed and purified TbHsp70.c and revealed for the first time that TbHsp70.c and Tbj2 can prevent thermal aggregation of malate dehydrogenase (MDH) and rhodanese and that Tbj2 stimulates the ATPase activity of TbHsp70.c. We report here that TbHsp70.c and Tbj2 have properties of molecular chaperones. A greater understanding of the Hsp70-Hsp40 partnerships is important as molecular chaperones have been implicated in parasite survival and growth.

## 2. Materials and Methods

### 2.1. Materials

Reagents used were purchased from Sigma Chemicals Co. (St. Louis, Mo USA), Merck Chemicals (Darmstadt, Germany), BioRad (USA), or Roche Molecular Biochemicals (Indianapolis, IN, USA). Nickel NTA resin was purchased from Pharmacia Biotech (Uppsala, Sweden). Anti-His antibody was purchased from GE Healthcare. Anti-TbHsp70.c was produced by and purchased from GenScript (USA). Plasmid TcHsp70B was a gift from Dr. D. Engman (Northwestern University Medical School, Chicago, USA). The *T. brucei TREU927* strain was a kind donation from Professor George Cross (Rockefeller University, New York, USA). *E. coli *strain BB1994 (MC4100* dnaK52 sidB1*::Tc pDMI,1::*CmR KanR*) was kindly provided by Dr. M. Mayer (Heidelberg University, Heidelberg, Germany).

### 2.2. Conservation Level of Cytoplasmic Hsp70 and Hsp40 Proteins from the Tritryps

**Figure 1:**
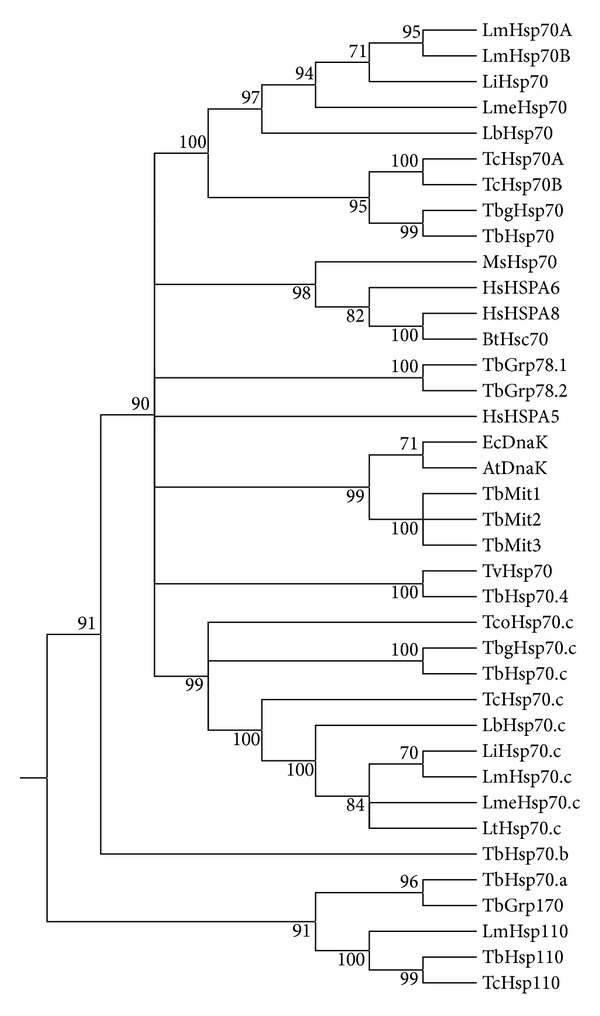
Phylogenetic analysis of Hsp70.c proteins from the TriTryps. Hsp70 amino acid accession numbers were obtained from GeneDB [41] and NCBI [42]. *L. braziliensis* Hsp70.c (GeneDB accession number LbrM.28.3030); *L. infantum* LiHsp70.c (GeneDB accession number LinJ.28.3040); LmHsp70.c; *L. Mexicana* LmeHsp70.c (GeneDB accession number LmxM.28.2820); *L. tarantole *LtHsp70.c (GeneDB accession number LtaP28.2840);* T. b. gambiense* TbgHsp70.c (GeneDB accession number Tbg972.11.12620); *T. congolense* TcoHsp70.c (GeneDB accession number TcIL3000.11.11940); TcHsp70.c (GeneDB accession number Tc00.1047053511211.220); LbHsp70 (GeneDB accession number LbrM.28.2990); LiHsp70 (GeneDB accession number LinJ.28.2950); TcHsp70A (GeneDB accession number Tc00.1047053511211.170); TcHsp70B (GeneDB accession number Tc00.1047053511211.160); LmHsp70A (GeneDB accession number LmjF28.2770); LmHsp70B (GeneDB accession number LmjF28.2780); LmeHsp70 (GeneDB accession number LmxM.28.2770); TbgHsp70 (GeneDB accession number Tbg972.11.12660); *T. vivax* Hsp70 (GeneDB accession number TvY486_0700470); TbHsp70 (GeneDB accession number Tb11.01.3110); TbHsp70.4 (GeneDB accession number Tb927.7.710); TbHsp110 (GeneDB accession number Tb10.389.0880); TbHsp70.c (GeneDB accession number Tb11.01.3080); TbHsp70.a (GeneDB accession number Tb09.160.3090); TbHsp70.b (GeneDB accession number Tb927.7.1030); TbGrp170 (GeneDB accession number Tb09.211.1390); TbGrp78.1 (GeneDB accession number Tb11.02.5500); TbGrp78.2 (GeneDB accession number Tb11.02.5450); TbMit1(GeneDB accession number Tb927.6.3740); TbMit2(GeneDB accession number Tb927.6.3750); TbMit3(GeneDB accession number Tb927.6.3800); HsHSPA5 (GenBank accession number AAI12964.1); HsHSPA6 (GenBank accession number NP_002146.2); HsHSPA8 (GeneDB accession number AAK17898.1); *Medicago sativa *Hsp70 (GenBank accession number AAV98051.1); EcDnaK (GenBank accession number BAA01595.1); AtDnaK (GenBank accession number AAR84665.1); *Bos Taurus *BtHsc70 (GenBank accession number P19120.2); LmHsp110 (GeneDB accession number LmjF18.1370); TcHsp110 (GeneDB accession number Tc00.1047053507831.60). A multiple sequence alignment was generated using the Muscle alignment tool and maximum likelihood analysis performed using MEGA5 version 5.2.2 [43]. The rooted tree was generated after 1000 bootstrap replicates, and confidence values are given on the branches.

Prior to phylogenetic tree analysis, trypanosomatid amino acid sequences were obtained from the GeneDB database [41] and other Hsp70 protein sequences were retrieved from the National Centre for Biotechnology Information (NCBI) [42], both sets of sequences in FASTA format. The accession numbers of all retrieved sequences are given in Figure 1. The multiple sequence analysis was performed using Muscle [44]. The phylogenetic tree was generated using Molecular Evolutionary Genetics Analysis (MEGA5) version 5.2.2 maximum likelihood analysis [43].

### 2.3. Construction of Expression Vectors pQE80-TbHsp70.c and pET28a-Tbj2

The coding regions of full length *TbHsp70.c* and *Tbj2 *were PCR amplified from *T. b. brucei TREU927 *genomic DNA. Forward primer (5′-GGT ACC ATG ACC TAC GAA GGA-3′) with a *Kpn*I restriction site (underlined) and reverse primer (5′-GTC GAC TTA CTC TGT GTT TGC-3′) with a *Sal*I restriction site (underlined) were used for *TbHsp70.c*. The forward (5′-GAATTCGGATCCCATATGGTGAAAGAAACAAAATAC-3′) (*EcoR*I, *BamH*I, *Nde*I) and reverse (5′-AAGCTTCTCGAGGTCGACCTATTGCTGCGTACACG-3′) (*Hind*III, *Xho*I, *Sal*I) primers were used for PCR amplification of *Tbj2.* The N-terminal His-tagged pQE80-TbHsp70.c and pET28a-Tbj2 constructs were verified by restriction digestion analysis and DNA sequencing (data not shown).

### 2.4. TbHsp70.c, TcHsp70B, and Tbj2 Protein Expression and Purification

An overnight culture was prepared by inoculating *E. coli* BB1994 [pQE80TbHsp70.c] in 25 mL 2 × YT broth supplemented with 100 µg/mL ampicillin and 50 µg/mL kanamycin at 30°C with shaking. The overnight culture was transferred to 225 mL 2 × YT broth and grown with shaking to midlog phase (A_600_ 0.6) before inducing TbHsp70.c protein expression by the addition of isopropylthiogalactoside (IPTG) to a final concentration of 1 mM. Cells were harvested (5000 g; 15 min) 3 hours after induction and resuspended in lysis buffer (100 mM Tris-HCl, pH 8, 300 mM NaCl, 10 mM imidazole). Cells were stored at −80°C overnight and thawed with the addition of 1 mg/L lysozyme and 1 mM PMSF culminating in lysis. Cellular debris was removed by centrifugation (12000 g; 40 min; 4°C) and the supernatant was incubated with nickel-charged sepharose beads in lysis buffer overnight at 4°C. The bead-suspension was centrifuged (5000 g; 1 min; 4°C) and three washes were performed with wash buffer (100 mM Tris-HCl, pH 8, 300 mM NaCl, 20 mM imidazole). Bound protein was eluted with 500 mM imidazole. Purified protein was subsequently dialysed overnight [100 mM Tris-HCl, pH8, 100 mM NaCl, 5% (v/v) glycerol, 50 mM KCl, 2 mM MgCl_2_, 0.5 mM dithiothreitol]. Purification of Tbj2 and TcHsp70B was adapted from the protocol carried out for TbHsp70.c. Both *E. coli* BL21(DE3) [pET28a-Tbj2] and BL21(DE3) [pET14b-TcHsp70B] cells were grown at 37°C, maintaining a selective pressure using kanamycin (50 µg/mL) and were harvested 5 hours after induction. To remove the presence of co-purified DnaK, Tbj2 and TcHsp70B bound to the Ni-NTA column were washed 5 times with the addition of 20% glycerol and 10 mM ATP to the wash buffer. All of other steps of the purification were performed in the same manner as for TbHsp70.c. Purified protein was quantified using Bradford's assay. Sodium-dodecyl sulphate-polyacrylamide gel electrophoresis (SDS-PAGE) analysis was used to evaluate the purity of the protein. Anti-TbHsp70.c was produced in rabbit using the C-terminal antigen, CQRGRGVTEGSGRPP at residues 648–662, from the TbHsp70.c protein sequence. Western analysis was used to verify the integrity and identity of TbHsp70.c using affinity-purified anti-TbHsp70.c peptide antibody. Western analysis was performed using anti-His antibody to target Tbj2 and TcHsp70B and anti-DnaK antibody to detect DnaK. Antibodies were detected using the ECL Advance Blotting Detection Kit and viewed using the Chemidoc EQ (BioRad, USA).

### 2.5. Aggregation Suppression Assays

#### 2.5.1. Suppression of Rhodanese Aggregation

The ability of TbHsp70.c and Tbj2 to suppress aggregation of bovine rhodanese (Sigma-Aldrich) was assessed spectrophotometrically. The assay was modified from [45]. Rhodanese was denatured overnight at 30°C; rhodanese denatured in denaturing buffer (6 M guanidine hydrochloride, 50 mM HEPES, pH 7.0, 100 mM NaCl) was added to assay buffer (50 mM HEPES, pH 7.0, 100 mM NaCl) to a final concentration of 1.5 µM was monitored at 300 nm over a period of 40 min at room temperature using a KC Junior microplate reader (Bio-Tek Instruments, USA). Molecular chaperone proteins of interest were added at various concentrations to measure their ability to suppress rhodanese aggregation. TcHsp70B was used as a positive control. TbHsp70.c, TcHsp70B, and Tbj2 are not aggregation prone as no significant increase in turbidity was observed when these proteins were assayed in the absence of rhodanese; protein samples that had been inactivated by boiling for 20 minutes also displayed no chaperone activity (data not shown). Absorbance was plotted as percent rhodanese aggregation over 40 min subsequent to normalizing against assays with rhodanese alone.

#### 2.5.2. Suppression of Malate Dehydrogenase (MDH) Aggregation

The abilities of TbHsp70.c and Tbj2 to prevent thermal aggregation of MDH were analysed by spectrophotometry. The assay was carried out as described by Botha and colleagues [46]. The assay was initiated by adding 0.72 µM MDH and the molecular chaperone proteins to assay buffer (50 mM Tris-HCl, 100 mM NaCl; pH 7.4) heated to 48°C. Aggregation of the protein substrate was monitored by measuring the scatter of light at 360 nm over 30 min at 48°C in a Helios Alpha DB spectrophotometer with a Peltier-controlled cell. Protein aggregation was assayed using varying concentrations of chaperone protein. The same controls as for the suppression of rhodanese assay were used.

### 2.6. ATPase Activity Assays

Hydrolysis of ATP by TbHsp70.c was assessed using a modified version of the ascorbic acid/ammonium molybdate colorimetric assay to measure the release of inorganic phosphate during the reaction [47]. The assay was performed as described in [37] with a few modifications. TbHsp70.c and control protein TcHsp70B (0.4 µM) were equilibrated to 37°C in ATPase buffer (25 mM HEPES, pH 7.4, 2 mM MgCl_2_, 50 mM KCl, 0.5 mM DTT). Stimulation of the basal ATPase activity of both TbHsp70.c and TcHsp70B by co-chaperone Tbj2 was assessed at equal concentrations (0.4 µM) and in a 2-fold molar excess to TbHsp70.c (1 µM) and TcHsp70B (1 µM). Samples were taken in triplicate at regular time intervals (0, 30, 60, 120, 180, and 240 minutes). All assays were corrected for the spontaneous breakdown of ATP observed in a control experiment in the absence of protein. Any background ATP hydrolysis observed for Tbj2 was corrected for by subtracting this activity from the reactions containing this protein.

### 2.7. Statistical Analysis

Statistical analysis was performed on data generated from both the aggregation suppression and ATPase activity assays. The relationship between two variables was analysed using one-way analysis of variance (ANOVA). Comparisons producing a *P* value < 0.05 were considered significant.

### 2.8. Detection of TbHsp70.c in *T. brucei * Lysates under Conditions of Heat Stress

Wild type Lister 927 variant 221 *T. brucei brucei *bloodstream form lysates (10^6^ cells/ml) were used for the heat stress inducibility experiment. Bloodstream form *T. brucei* Lister 927 variant 221 strain trypanosome parasites were cultured in filter sterilized complete Iscoves Modified Dulbeccos Media (IMDM) based HM1-9 medium [IMDM base powder, 3.6 mM sodium bicarbonate, 1 mM hypoxanthine, 1 mM sodium pyruvate, 0.16 mM thymidine, 0.05 mM bathocuprone sulphate acid, 10% (v/v) heat inactivated Foetal Bovine Serum, 1.5 mM L-cysteine, 0.2 mM *β*-mercaptoethanol, pH 7.5] in a humidified chamber at 37°C with an atmosphere of 5% CO_2_. Separate 25 ml culture of cells were exposed to heat shock at 42°C for a period of 60 min in plugged flasks, allowing no entry of CO_2_. A control experiment was performed under the same conditions maintaining the temperature at 37°C. Cell lysates were harvested by centrifugation at 800 g for 10 min, washed twice in PBS buffer (10 mM Na_2_HPO_4_, 2 mM KH_2_PO_4_, 137 mM NaCl, 2.7 mM KCl), and repelleted prior to resuspension in SDS-PAGE loading buffer to a final cell count of 5 × 105 cells/*μ*L. The lysates (5 × 10^6^ cells per lane) were resolved on a 10% SDS-PAGE gel. Differences in TbHsp70.c protein expression were detected using polyclonal rabbit anti-TbHsp70.c peptide antibody (1 : 5000) and goat anti-rabbit IgG HRP-conjugated secondary antibody (1 : 5000) in subsequent western analysis.

## 3. Results

Bioinformatic analysis revealed *T. brucei* Hsp70.c to be a eukaryotic isoform that may represent a novel family of Hsp70 proteins as it did not cluster phylogenetically with any of the other primary Hsp70 proteins [24]. Phylogenetic tree analysis was used to establish orthology between the novel Hsp70.c proteins within the TriTryps and to compare their phylogenetic relationships with cytoplasmic Hsp70s from the TriTryps and the Hsp70s from the *T. brucei* complement along with well-characterized eukaryotic and prokaryotic canonical Hsp70 proteins (Figure 1). TbHsp70.c may prove to be an anti-parasitic drug target as phylogenetic analysis suggests that TbHsp70.c clusters only with Hsp70 proteins from the Tritryps. Not surprisingly, the Hsp70.c proteins clustered together phylogenetically, TbHsp70.c formed a monophyletic clade with TbgHsp70.c and a close link with TcoHsp70.c, suggesting possible paralogy. The Hsp70.c cluster formed a closer phylogenetic relationship with the cytoplasmic Hsp70s, including inducible human Hsp70B (HsHSPA6), constitutively expressed human Hsc70 (HsHSPA8), bovine Hsc70 and plant Hsp70 than with mitochondrial Hsp70s, the NEF-acting Hsp110s, or the DnaKs (prokaryotic Hsp70 homologue) (Figure 1).

**Figure 2:**
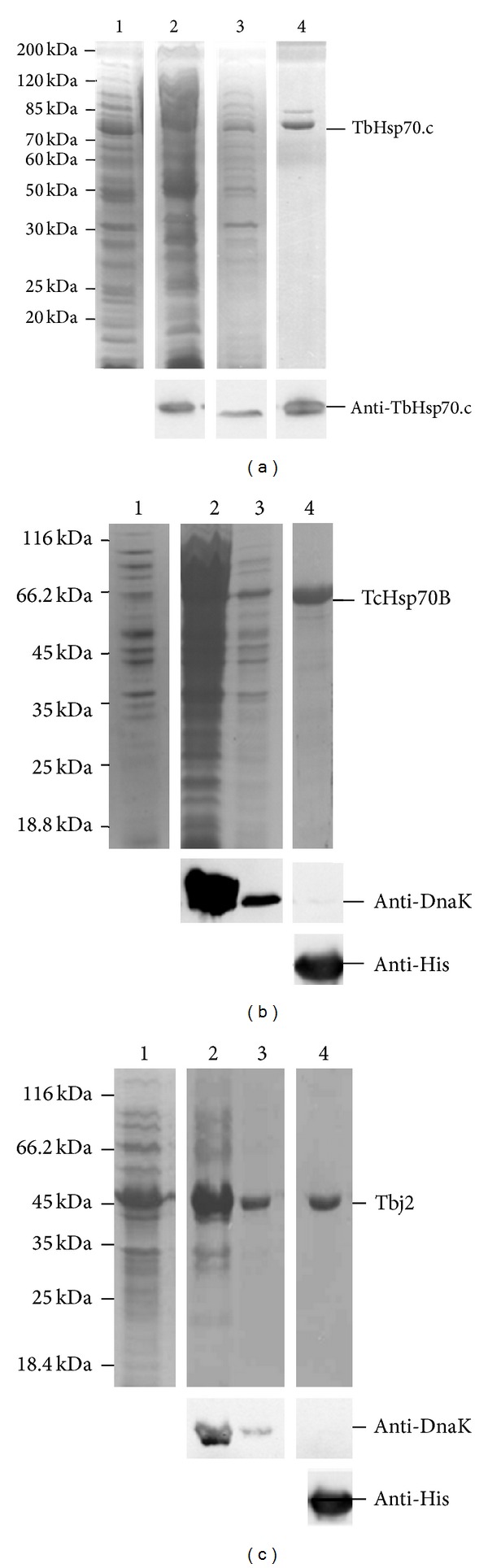
Expression and purification of recombinant TbHsp70.c, TcHsp70B, and Tbj2. (a) SDS-PAGE of a TbHsp70.c purification. *Upper panel: Lanes 1*- *E. coli* BB1994 [pQE80TbHsp70.c] whole lysate 3 hr post IPTG induction, *2 - *fraction unbound to Ni-NTA column, *3*- wash using 20 mM imidazole, *4 - *first TbHsp70.c elution (500 mM imidazole).  *Lower panel:* western analysis of TbHsp70.c at 73 kDa using anti-TbHsp70.c peptide antibody. (b) SDS-PAGE analysis of a TcHsp70B purification. *Upper panel: Lanes 1* - *E. coli* BL21(DE3) [pET14bTcHsp70B] whole cell lysate 5 hours post IPTG induction, *2* - fraction unbound to Ni-NTA column, *3* - wash using 20 mM imidazole, 20% glycerol and 10 mM ATP, *4* - TcHsp70B first elution (500 mM imidazole).  *Second panel: *western analysis of copurified DnaK at 70 kDa using anti-DnaK antibody. *Third panel:* TcHsp70B detected at 70 kDa by western analysis using anti-His antibody. (C) SDS-PAGE analysis of a Tbj2 purification. *Upper panel: Lanes 1* - *E. coli* BL21(DE3) [pET28aTbj2] whole cell lysate 5 hours post IPTG induction, *2* - fraction unbound to Ni-NTA column, *3* - wash containing 20 mM imidazole, 20% glycerol and 10 mM ATP, *4* - Tbj2 first elution (500 mM imidazole).  *Second panel: *western analysis of co-purified DnaK at 70 kDa using anti-DnaK antibody. *Third panel:* Tbj2 detected at 44 kDa by western analysis using anti-His antibody. Numbers to the left of the panels indicate the protein marker ladder, the Peqlab peqGOLD Protein Marker II in (a) and the Fermentas Pierce Unstained Protein MW Marker in (b) and (c).

Recombinant TbHsp70.c was expressed and purified from an *E. coli dnaK*-minus strain (Figure 2(a)). The *E. coli dnaK*-minus strain was used to eliminate endogenous DnaK, the bacterial homologue of Hsp70. Soluble TbHsp70.c was successfully purified by nickel-affinity chromatography under non-denaturing conditions and anti-TbHsp70.c peptide antibodies recognized full-length TbHsp70.c at 73 kDa, confirmed by western analysis (Figure 2(a)). 600 mg of protein was purified from 1 L of *E. coli *cells and was of sufficient purity for subsequent *in vitro* assays. An alternative strategy was adopted for the purifications of recombinant TcHsp70B and Tbj2 to eliminate co-purifying DnaK due to a lack of an *E. coli dnaK*-minus strain compatible with the pET vectors. Modifications to the nickel-affinity chromatography protocol included the addition of 20% glycerol and 10 mM ATP to the wash steps. The non-denaturing purification resulted in the removal of co-purified DnaK, confirmed by western analysis, from both the TcHsp70B (Figure 2(b)) and Tbj2 (Figure 2(c)) purifications. For the TcHsp70B and Tbj2 purifications, 1176 mg and 900 mg of protein were purified from 1 L of *E. coli* cells, respectively, and the elimination of DnaK warranted the use of TcHsp70B and Tbj2 in subsequent *in vitro* assays.

**Figure 3:**
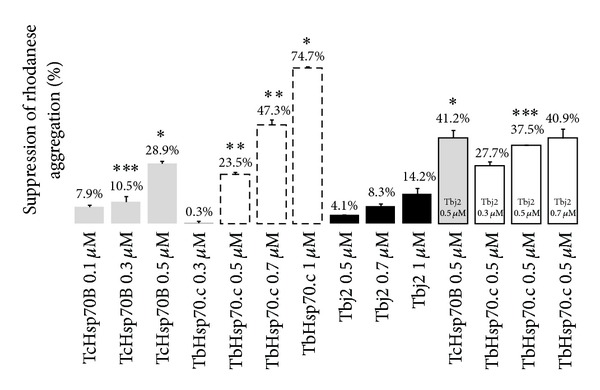
Suppression of rhodanese aggregation by TbHsp70.c, TcHsp70B, and Tbj2. The rhodanese concentration was maintained at 1.5 µM, the aggregation of denatured rhodanese was measured at 300 nm over 40 min and the concentrations of all components are given in the figure. TcHsp70B, TbHsp70.c, and Tbj2 suppress aggregation of denatured rhodanese in a dose-dependent manner. Each assay was conducted in triplicate and three independent experiments on independent batches of protein were conducted and the data shown represents that of a typical experiment. The bars represent standard deviations. *P < 0.001, **P < 0.01, and ***P ≤ 0.05.

TbHsp70.c was able to suppress the protein aggregation of chemically denatured rhodanese in a dose-dependent manner (Figure 3). TcHsp70B, an orthologue of *Bos taurus* HSPA8, was used as a positive control. In addition to demonstrating typical chaperone properties [32, 34], the previously characterized TcHsp70B, also homologous to TbHsp70, contains an intact C-terminal EEVD motif. The addition of TbHsp70.c and TcHsp70B resulted in a marked increase of aggregation suppression in a dose-dependent manner (Figure 3). Tbj2 (0.5 µM, 0.7 µM, 1 µM) resulted in 4.1%, 8.3%, and 14.2% suppression of rhodanese aggregation, respectively. To investigate the ability of Tbj2 to enhance the holdase function of TbHsp70.c, a fixed concentration of TbHsp70.c was used and Tbj2 concentrations were varied. The addition of Tbj2 at a submolar concentration to TbHsp70.c resulted in 27.7% suppression of rhodanese aggregation (Figure 3). Equal concentrations of Tbj2 and TbHsp70.c resulted in 37.5% suppression of rhodanese aggregation, which was comparable to the 41.2% suppression produced by TcHsp70B at equimolar concentrations to Tbj2. Tbj2 in partnership with TbHsp70.c demonstrated increased aggregation suppression. At equimolar concentrations Tbj2 demonstrated a synergistic effect and thus the ability to function as a co-chaperone to both TcHsp70B and TbHsp70.c.

**Figure 4:**
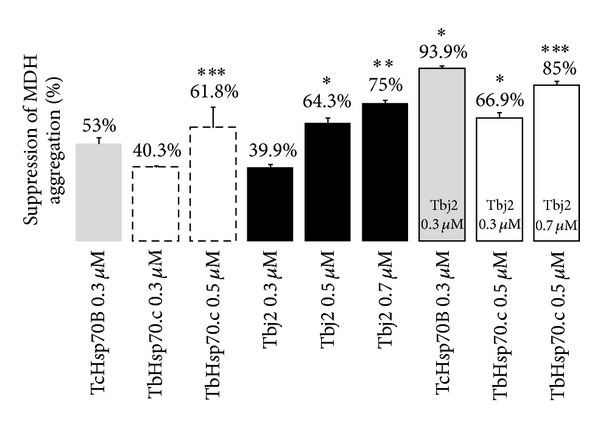
Suppression of MDH aggregation by TbHsp70.c, TcHsp70B and Tbj2. The reaction was initiated by the addition of MDH (0.715 µM) to the assay buffer at 48°C; the concentrations of all components are given in the figure. TcHsp70B suppresses aggregation of MDH. TbHsp70.c and Tbj2 both suppress aggregation of MDH in a dose-dependent manner. The effect of Tbj2 on the ability of TbHsp70.c and TcHsp70B to suppress MDH aggregation was investigated. Each assay was conducted in triplicate and three independent experiments on independent batches of protein were conducted and the data shown represents that of a typical experiment. The bars represent standard deviations. *P < 0.001, **P < 0.01 and ***P < 0.05.

MDH was used as a second substrate to establish whether TbHsp70.c and Tbj2 could suppress aggregation of MDH. BSA was used as a negative control and had no effect on MDH aggregation (data not shown). TcHsp70B resulted in 53% suppression of MDH aggregation (Figure 4). A dose response was evident upon the addition of various concentrations of TbHsp70.c as turbidity levels decreased with an increase in chaperone concentration (Figure 4). A similar result was obtained for Tbj2 where increased chaperone concentrations caused decreased turbidity and a dose-dependent suppression of MDH aggregation (Figure 4). The ability of Tbj2 to enhance the holdase function of TbHsp70.c was assessed by maintaining constant TbHsp70.c concentrations and varying those of Tbj2 (Figure 4). TbHsp70.c (0.5 µM) resulted in 61.8% suppression of MDH aggregation (Figure 4). The addition of Tbj2 at a submolar concentration (0.3 µM) to TbHsp70.c resulted in 66.9% MDH aggregation suppression and Tbj2 at molar excess (0.7 µM) resulted in 85% suppression of MDH aggregation (Figure 4). Tbj2 concentrations of 0.3 µM and 0.7 µM enhanced TbHsp70.c chaperone activity; however, the increase in turbidity was not enough to be an additive effect. Equal concentrations of Tbj2 and control protein TcHsp70B resulted in 93.9% suppression of MDH aggregation, which appeared to be an additive effect (Figure 4). Tbj2 did not demonstrate the ability to co-chaperone either TbHsp70.c or Tchsp70B using MDH as a substrate. Tbj2 may have been binding as substrate to TbHsp70.c, thus resulting in the decreased suppression of MDH aggregation that was observed.

**Figure 5:**
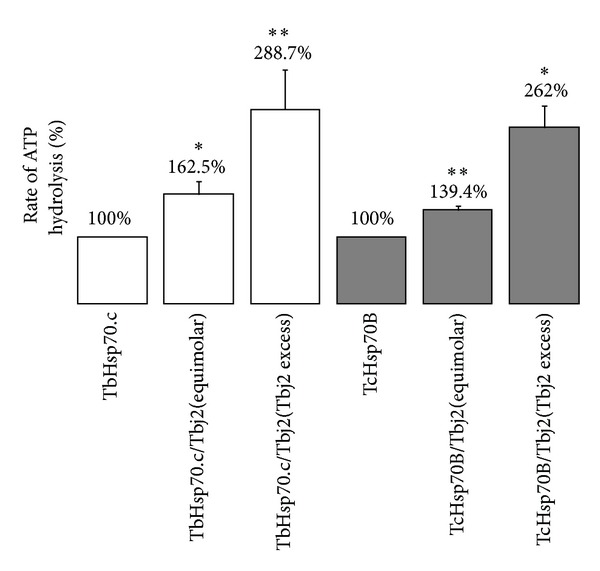
Stimulation of TbHsp70.c and TcHsp70B ATPase activity by co-chaperone Tbj2. The ATPase activity of TbHsp70.c (0.4 µM) and TcHsp70B (0.4 µM) was assayed in the absence and presence of Tbj2 at equimolar concentrations. Tbj2 was assayed in a 2-fold molar excess to TbHsp70.c (1 µM) and TcHsp70B (1 µM). The unstimulated ATPase activity of TbHsp70.c and TcHsp70B is represented by 100%. The concentration of ATP, present in all reactions, was maintained at 600 µM. The data points were determined from triplicate ATPase activity measurements for three independent batches of purified protein and the data shown represents that of a typical experiment. The bars represent standard deviations.*P < 0.01 and **P < 0.05.

The ability of Tbj2 to stimulate the basal ATPase activity of TbHsp70.c and TcHsp70B was investigated. TbHsp70.c was determined to have a basal ATPase activity of 7.6 nmol Pi/min/mg and TcHsp70B a basal ATPase activity of 18.3 nmol Pi/min/mg. The basal ATPase activity of TbHsp70.c and TcHsp70B was represented as 100% (Figure 5). Equal concentrations of Tbj2 and TbHsp70.c resulted in a significant 1.62-fold stimulation of the basal ATPase activity of TbHsp70.c (Figure 5). Equal concentrations of Tbj2 and TcHsp70B caused a slightly lower but still significant 1.39-fold stimulation of TcHsp70B basal ATPase activity (Figure 5). Tbj2 at 2-fold molar excess to TbHsp70.c and TcHsp70B resulted in a significant 2.88-fold enhancement of the basal ATPase activity of TbHsp70.c and a significant 2.62-fold stimulation of TcHsp70B ATPase activity (Figure 5). TbHsp70.c, TcHsp70B, and Tbj2 denatured by boiling displayed no ATPase activity (data not shown).

**Figure 6:**
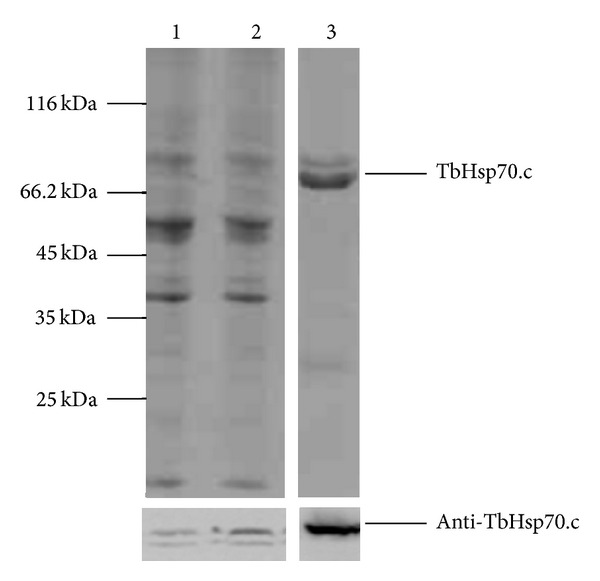
TbHsp70.c expression is enhanced under heat stress. Detection of TbHsp70.c expression levels in a whole cell lysate prepared from Lister 927 *T. b. brucei *v221 cells in the absence (37°C) and presence (42°C) of heat stress by 10% SDS-PAGE (5 × 10^6^ cells/lane); western analysis revealed that TbHsp70.c is heat inducible. *Upper panel: Lanes 1* - cells incubated for 1 hour at 37°C; *2* – cells incubated for 1 hour at 42°C; *3* – purified recombinant TbHsp70.c in *E. coli* BB1994 [pQE80-TbHsp70.c]. *Lower panel: Lanes 1*–*3* - detection of target protein by western analysis using anti-TbHsp70.c antibody, showing increased expression levels of TbHsp70.c under heat stress. The experiment was performed in triplicate using three different whole cell lysates and the figure represents the findings of a typical experiment.

Under conditions of stress, a cell responds by increasing the synthesis of heat shock proteins to manage the elevated levels of denatured proteins. *T. b. brucei 427 *V221 cells were exposed to heat shock by incubating them at 42°C for one hour. Recombinant TbHsp70.c purified from *E. coli* BB1994 cells was used as a positive control. Even though there was no notable difference in the TbHsp70.c protein levels on the SDS-PAGE gel, TbHsp70.c protein expression proved to be inducible by heat stress, as shown by an increase in the top band in the western analysis (Figure 6). Two bands were detected by the anti-TbHsp70.c peptide antibody. The top band at 73 kDa increased in intensity from 37°C to 42°C indicating that at 37°C TbHsp70.c was expressed in bloodstream form cells and upon exposure to heat stress TbHsp70.c was upregulated. Interestingly, the expression level of the non-specific 68 kDa band, detected by anti-TbHsp70.c peptide antibodies, remained constant upon heat shock, suggesting it to be a separate protein and not a degradation product of TbHsp70.c (Figure 6).

## 4. Discussion

A study of the evolutionary relationships between kinetoplastid Hsp70 proteins and typical Hsp70 proteins has revealed a novel and divergent group of proteins, the orthologues TbHsp70.c, TcHsp70.c, and LmHsp70.c. This is the first report of the successful expression and purification of TbHsp70.c. Tbj2 was selected as a probable co-chaperone of TbHsp70.c due to the presence of its functional J-domain and because of its predicted cytosolic localization (data not shown). When studying molecular chaperones, the presence of DnaK is a major concern due to the possibility that basal expression of bacterial or co-purified DnaK could mask the chaperone activity of the *T. brucei* heat shock proteins under investigation. The co-purification of DnaK is likely as a result of denatured exposed surfaces of the target protein interacting with DnaK by acting as a substrate. Using an *E. coli dnaK*-minus strain and modifications to the purification protocol, DnaK was eliminated from the TbHsp70.c and the Tbj2 and TcHsp70B purifications, respectively.

Amongst the various roles of Hsp70 proteins is the ability to act as holdases, preventing aggregation of proteins [6]. Demonstration of the holdase-function, by suppressing protein aggregation, is used to display chaperone activity [48, 49]. MDH aggregation suppression assays are often performed in the absence of ATP [50, 51]. The addition of ATP decreases the affinity of an Hsp70 protein for MDH, resulting in its release and subsequent re-aggregation [52]. A loss of affinity for substrate in the presence of ATP has been demonstrated for *P. falciparum* Hsp70 [53], *E. coli*Hsc66, DnaK [54] and bovine Hsp90 [55]. The model thermolabile substrate MDH was used in this study to investigate the ability of TbHsp70.c and Tbj2, in isolation and in partnership, to suppress the heat-induced aggregation of MDH. A second substrate, rhodanese, was selected to investigate whether the substrate binding specificity of TbHsp70.c for rhodanese would differ considering the presence of fewer hydrophobic residues within rhodanese than in MDH. A recent *in silico *study using an *E. coli *DnaK L484W mutant predicted that an increase in hydrogen bonds and hydrophobic interactions allows an enhanced interaction between chaperone and substrate [56]. TbHsp70.c was shown to bind and suppress aggregation of both rhodanese and MDH, thereby displaying typical chaperone activity. However, TbHsp70.c suppressed aggregation of MDH to a greater degree than was observed for rhodanese.

Hsp70s generally function in cooperation with Hsp40s, where Hsp40 either mediates the interaction of Hsp70 with its substrate or “holds” the substrate and recruits Hsp70 to the unfolded polypeptide [57]. Hsp40 proteins are able to bind unfolded substrates independently of Hsp70 proteins and prevent their aggregation [18, 58]. Once bound, the substrate is transferred to Hsp70 by the J-protein; at the same time Hsp40 stabilizes the Hsp70-substrate association by stimulating the ATPase activity of Hsp70 [58]. Tbj2 successfully suppressed aggregation of MDH. A similar result was seen for the homologue of Tbj2, Tcj2, in its ability to independently suppress aggregation of MDH [37]. However, Tbj2 showed a considerably decreased ability to suppress rhodanese aggregation. The reduced chaperone activity observed in this study using rhodanese could furthermore be as a result of non-productive and aggregation-prone intermediates formed whilst rhodanese refolds [59]. In the presence of TbHsp70.c, Tbj2 appeared to enhance the suppression of aggregation of rhodanese. The effect of Tbj2 on both TbHsp70.c and TcHsp70B was additive rather than synergistic when using MDH. A similar result was observed during the suppression of aggregation of MDH by *Plasmodium falciparum* Hsp40 and Hsp70 [51]. The ability of a Type III Hsp40 protein, Tbj1, to function as a co-chaperone was supported by the findings that Tbj1 assisted *Trypanosoma cruzi* Hsp70 and *Medicago sativa *Hsp70 in suppression of MDH aggregation [37]. Suppression of protein aggregation assays has also been used to successfully determine the effect of small molecule modulators on the abilities of PfHsp70 proteins to suppress the aggregation of alcohol dehydrogenase [60] and MDH [50]. The ability of TbHsp70.c to successfully suppress aggregation of two individual substrates was not surprising. Even though some conservation has been lost in the TbHsp70.c hydrophobic arch, some residues are replaced by acidic and aliphatic residues and the hydrophobic pocket, represented by the valine residue, is well conserved (data not shown).

Enhanced Hsp70 ATPase activity is specific and is modulated through the interaction of an Hsp40 partner. Tbj2 in excess stimulated the ATP hydrolysis activity of TbHsp70.c approximately 3-fold. This data would suggest that Tbj2 acts as a co-chaperone by means of its J-domain interacting with the ATPase domain of TbHsp70.c, either recruiting substrates to the Hsp70 or merely mediating the interaction of the Hsp70 with its substrate and stimulating the ATPase activity of the Hsp70. However, the level of ATPase activity stimulation by Tbj2 is low in comparison to what has previously been observed for Hsp70-Hsp40 partnerships. Yeast Hsp40, Ydj1p, was shown to stimulate the ATPase activity of yeast Hsp70, Ssa1p, by 6-8-fold [18, 61, 62]. Tbj2, homologue of Tcj2, demonstrated a greater stimulation of TbHsp70.c ATPase activity than was observed for Tcj2-stimulated ATPase activity of TcHsp70B [34]. The low stimulation of the ATPase activity of TcHsp70 by Tcj2 was suggested to be as a result of a masking effect of the already very high basal ATPase activity of TcHsp70B, reported to be 100 times greater than that observed for human Hsp70 [34]. Further characterization of the ATPase activity of TbHsp70.c would entail investigating the activation of the ATPase activity of TbHsp70.c by various substrates. Maximal stimulation of the ATPase activity of Hsp70 proteins has been demonstrated to take place in the presence of both co-chaperone and substrate [63].

To date, very little research has been performed on the heat shock response within *T. brucei*. The focus has been on investigating the effect of heat stress on *T. cruzi* and/or its vector, *Panstrongylus megistus* [64] as well as on molecular chaperones belonging to the *T. cruzi* parasite [65]. A study in which *T. b. brucei* bloodstream form cultures were exposed to heat shock resulted in the upregulation of the expression levels of TbHsp70.c. This finding would imply that TbHsp70.c is required for protein quality control not only under normal conditions but also when the cell is placed under thermal stress. This is the first report of data showing that the expression levels of TbHsp70.c are increased by heat stress. TcHsp70 mRNA levels showed a very significant four-fold upregulation upon heat stress at 37°C when compared to mRNA levels at 28°C [65].

## 5. Conclusions

The ability of Tbj2 to significantly enhance the basal ATPase activity of TbHsp70.c suggests that Tbj2 functions as a co-chaperone of TbHsp70.c. However, further experimental analyses including binding studies and co-localization will need to be performed to conclusively confirm Tbj2 as co-chaperone of TbHsp70.c. Furthermore, the possibility of other cytoplasmic Tbj proteins functioning as co-chaperones of TbHsp70.c will need to be investigated. Due to its atypical features, TbHsp70.c is likely to interact with a specific set of protein substrates in the cell. TbHsp70.c is the first *T. brucei* Hsp70 protein to be functionally characterized, along with its probable partnership with co-chaperone Tbj2. This research opens up prospects for further studies of the yet unexplored multiple Hsp70/Hsp40 partnerships in *T. brucei*. Future research would entail isolating and characterizing the remaining 11 Hsp70 proteins from *T. brucei* to enable differentiation between unique and canonical features and furthermore investigating which of them form partnerships with the 65 Hsp40 proteins. Characterization of these and other chaperone/co-chaperone interactions could further enhance understanding of the cell biology of *T. brucei*.
